# Sexually Transmitted Infections Among Kenyan Adolescent Girls and Young Women With Limited Sexual Experience

**DOI:** 10.3389/fpubh.2020.00303

**Published:** 2020-07-14

**Authors:** Tiffany Yuh, Murugi Micheni, Stacy Selke, Lynda Oluoch, Catherine Kiptinness, Amalia Magaret, Bhavna Chohan, Kenneth Ngure, Anna Wald, Nelly R. Mugo, Alison C. Roxby

**Affiliations:** ^1^Department of Medicine, University of Washington, Seattle, WA, United States; ^2^Centre for Clinical Research, Kenya Medical Research Institute, Nairobi, Kenya; ^3^Department of Laboratory Medicine, University of Washington, Seattle, WA, United States; ^4^Department of Global Health, University of Washington, Seattle, WA, United States; ^5^Centre for Virus Research, Kenya Medical Research Institute, Nairobi, Kenya; ^6^Department of Community Health, Jomo Kenyatta University of Agriculture and Technology, Nairobi, Kenya; ^7^Vaccine and Infectious Disease Division, Fred Hutchinson Cancer Research Center, Seattle, WA, United States

**Keywords:** adolescent, sexually transmitted infection, chlamydia, bacterial vaginosis, Africa

## Abstract

**Objectives:** Globally, the highest rates of sexually transmitted infections (STIs) are among the 15-24 age group. Studying adolescent girls and young women (AGYW) pre-sexual debut could identify risk factors for STI acquisition.

**Methods:** We recruited a prospective cohort of low-risk AGYW aged 16-20 in Kenya. Participants were HIV and HSV-2 seronegative and reported no history of sexual intercourse or reported sex with one partner. Participants underwent genital exams, nucleic acid testing of vaginal swabs for *Neisseria gonorrhea* (NG), *Chlamydia trachomatis* (CT), *Trichomonas vaginalis* (TV), and vaginal gram stains for vaginal dysbiosis by Nugent score. STI correlates were described using χ^2^ test and *t*-test.

**Results:** We enrolled 400 AGYW, of which 322 (80.5%) reported never having had sex, while 78 (19.5%) reported prior sex with 1 partner. Among the 78 participants reporting prior sex, 20 (25.6%) reported contraception use in the last 3 months, with 60% using only emergency contraceptive pills. Despite self-reported history, of 373 subjects who underwent STI testing, 49 subjects (13.1%) tested positive for STIs, with 41 CT, 5 GC, and 3 TV cases. Of these 49 subjects, 33 (67.3%) reported no prior sexual intercourse. Bacterial vaginosis was rare and 90% of subjects had a normal Nugent score (0–3).

**Conclusions:** Upon baseline evaluation of a cohort of low risk AGYW, we found high numbers of STIs, especially CT, which is not routinely screened for in Kenyan settings. Interventions to address STIs and unintended pregnancy should target girls pre-sexual debut, including those who do not self-identify as at risk.

## Introduction

The World Health Organization estimates there were 376 million new cases of four curable sexually transmitted infections (STIs), chlamydia, gonorrhea, syphilis, and trichomoniasis, in 2016 (1). Globally, the highest reported rates of STIs are among youth ages 15-24 (2), especially in resource-poor countries (3). There are few large-scale studies about STI prevalence in low- and middle-income countries, but several studies of populations in sub-Saharan Africa note a higher prevalence of STIs in younger women than in older women, as well as in women over men (4, 5). The high prevalence of STIs observed among adolescent girls and young women (AGYW) compared to their male counterparts may be related to increased biological susceptibility, decreased educational, and economic opportunities, older sexual partners, increased risk for sexual coercion, and cultural norms and gender inequalities that reduce access to sexual health resources (6). The global STI epidemic will increase the burden of long-term health effects of undiagnosed and untreated disease, including pelvic inflammatory disease, infertility, ectopic pregnancy, and increased risk of HIV acquisition (1).

Few studies have followed adolescents longitudinally from pre-sexual debut. To understand risk factors for STI acquisition present from early adolescence, we enrolled a cohort of AGYW in Kenya to follow them as they transition from virginal to sexually active. We report baseline STI data from the study enrollment visit.

## Methods

### Study Design, Recruitment and Procedures

We recruited a cohort of AGYW aged 16-20 in Thika, Kenya between 2014 and 2016. Thika is located on hour from Nairobi and residents live in a suburban setting. A community advisory group composed of individuals with positions of influence within the community (including school principals, peer counselors, health care workers, district chiefs/administrators, religious leaders, women's groups) assisted with community engagement by introducing the study staff as health care workers conducting a research study with the aim of teaching young girls about reproductive health and preventing HSV-2. For those interested in the study, girls aged 16-17 were accompanied by their guardians to hear a health presentation, and if they desired participation, subsequently screened for the study. Those aged ≥18 years came independently for screening. We initially sought AGYW who reported no prior history of sexual intercourse. The protocol was subsequently amended to include those who reported only a single sex partner. Sex was defined as vaginal penetrative intercourse. The eligibility criteria were not divulged to potential study participants or to individuals outside of the study; parents/guardians did not learn whether their child met eligibility criteria. Potential participants needed to be willing to undergo external genital examinations and remain in follow-up for 3 years. Guardians who consented to study screening and participation for girls <18 years were informed that, after enrollment, all interactions between participants and study staff would remain confidential.

After consent was obtained, HIV and HSV-2 ELISA testing were performed, and demographic history was elicited. AGYW were excluded if HIV or HSV-2 positive. Qualifying participants returned within 3 weeks for an enrollment visit, at which time a female clinician performed an external genital exam, and obtained vaginal swab specimens for testing for *Neisseria gonorrhea* (GC), *Chlamydia trachomatis* (CT), *Trichomonas vaginalis* (TV), and bacterial vaginosis (BV). No testing was performed for syphilis because prior studies at the same site had found extremely low rates of syphilis, including among high risk populations, such that positive tests would be more likely to be false positives. Participants reporting prior sex completed a sexual history questionnaire. Information and samples were coded to protect confidentiality.

Participants were notified of any relevant study results directly and confidentially by study staff, and were treated for any identified STIs.

### Ethics, Consent, and Permissions

Study approval was obtained from the Kenya Medical Research Institute Scientific Ethics Review Unit, and the University of Washington Institutional Review Board. For participants under age 18, written informed assent was obtained, and written informed consent obtained from a parent/guardian. Assent was obtained separately and privately from parents/guardians, to allow participants to ask questions and to decide whether they wanted to participate free of parental influence. For participants age 18 or older, written informed consent was obtained. Participants received free, confidential medical services including contraception, HIV testing and condoms, and psychological counseling services. Participants also received transportation reimbursement for each study visit attended.

### Laboratory Methods

At the screening visit, a rapid HIV test was performed using whole blood from a finger prick, with the Determine HIV-1/2 Rapid test (Abbott, Chicago, Illinois). The Uni-Gold Recombigen HIV-1/2 (Trinity Biotech, Wicklow, Ireland) was used as a confirmatory test, performed at the Paediatrics Lab at Kenyatta National Hospital. At follow-up visits, blood was collected for HIV ELISA testing using Vironostika® HIV Uni-Form II Ag-Ab (Biomerieux, Marcy-l'Etoile, France). If the HIV test was positive, the participant was referred to the nearest HIV Comprehensive Care Clinic for follow-up. At the screening visit, a blood sample was sent to Kenyatta National Hospital for HSV-2 testing using the HerpeSelect HSV-2 ELISA test (Focus Diagnostics, Cypress, CA). For sample collection for GC, CT, and TV testing, a dacron swab was placed in the vagina (no speculum was used). The swab was shipped to the University of Washington Center for AIDS Research laboratory in Mombasa, Kenya for testing. Nucleic acid amplification testing was performed using the Gen-Probe APTIMA test (Hologic, Marlborough, MA). A vaginal swab was also collected for BV testing. The swab was used to create a Gram stain, and the slide was transported to Kenyatta National Hospital for evaluation by Nugent's criteria (7). A Nugent score ≥7 was defined as positive for BV.

### Statistical Analysis

An STI was defined as chlamydia, gonorrhea, or trichomoniasis by NAAT testing. The study was powered for HSV-2 acquisition. Based on HSV-2 prevalence data (8), projected incidence of 7.8% per year, and a median age of sexual debut of 18 (9), we assumed that enrolling 400 HSV-2 seronegative persons would result in 73 observed HSV-2 acquisitions, accounting for 13% dropout.

Data were entered into a password-secured REDCap database (10). Descriptive statistics were used to characterize the cohort. We examined whether STI positivity was associated with education, socioeconomic status, sexual history, and physical exam findings. χ^2^ test and Fisher's exact test were used to compare categorical variables, and *t*-test for independent samples for continuous variables. A multivariate logistic regression model was constructed, adjusting for possible cofactors of STI (age, BV, sexual activity, education level, rural residence, vaginal discharge). Each cofactor was added stepwise to the model and included in the final model if significantly associated (*p* <0.1). Data were analyzed using Stata version 15.1 (StataCorp, College Station, Texas); statistical significance was defined as 2-sided *p* value <0.05. Missing data was left out of the analysis.

## Results

### Baseline Characteristics

**Figure 1:**
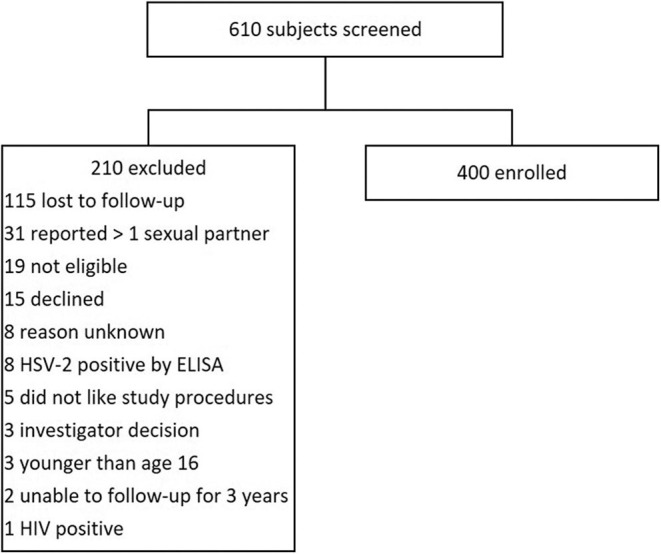
Flow diagram for enrollment.

We screened 610 AGYW, and 400 were enrolled. Two of the most common reasons for ineligibility were failure to return to clinic or report of more than one sexual partner (Figure 1). The median age of participants at enrollment was 18.6 years (IQR 17.6-19.4) and the median years of schooling was 12 (IQR 10-12) (Table 1). There were 155 (38.8%) AGYW residing in an urban setting. Within the home, 191 (47.8%) had running water, 290 (72.5%) had concrete floors, and 277 (69.3%) had electricity. Among enrolled participants, 322 (80.5%) reported no prior history of sex, while 78 (19.5%) reported one lifetime sexual partner.

**Table 1:** Baseline characteristics of adolescent girls and young women enrolled in the prospective cohort.

**Characteristic**	**All subjects *N* = 400**
Age	18.6 (17.6-19.4)
Years of school completed	12 (10-12)
Has source of income	151 (37.8)
Monthly income (Ksh)	0 (0-1000)
Urban residence	155 (38.8)
Resides in formal settlement	302 (75.5)
Running water	191 (47.8)
Concrete floor	290 (72.5)
Electricity	277 (69.3)
Tile/metal roof	382 (95.5)
Number of rooms in house	3 (2-4)
Number of people in house	5 (4-6)
No prior sex	322 (80.5)
One lifetime sexual partner	78 (19.5)

*Data are expressed as median (IQR) or n (%). Ksh, Kenyan shilling*.

### Sexual History

Among the 78 participants who reported one lifetime sexual partner, the median age at first sexual intercourse was 18.7 years, and the median age of the sexual partner was 22 (IQR 21-24) (Table 2). Seventy (89.7%) of these participants endorsed sex with consent, and 20 (25.6%) reported receiving a reward for sex. Twenty (25.6%) AGYW reported contraception use in the last 3 months, with most (60%) reporting emergency pill use, and 35% reporting condom use.

**Table 2:** Sexual history for adolescent girls and young women reporting prior sexual intercourse (*N* = 78).

**Characteristic**	***N* = 78**
Age at first sex	18.7 (18.1-19.3)
Condom use at first sex	49 (62.8)
Age of first sexual partner	22 (21-24)
Sex with consent	70 (89.7)
Reward/money for sex	20 (25.6)
Number of times sex in last 3 months	1 (0-2)
Contraception use in last 3 months	20 (25.6)
Oral	2 (10)
IUD	1 (5)
Injectable	1 (5)
Implant	0 (0)
Emergency pill	12 (60)
Condom	7 (35)

*Data are expressed as median (IQR) or n (%)*.

### Clinical Findings

Overall, 376 (94%) AGYW had external genital exams done, and 4 were circumcised. Eighteen (4.8%) of AGYW were noted to have an abnormal genital finding, with 11 participants (3%) having abnormal vaginal discharge. Of these 11 participants, 4 tested positive for an STI. Other abnormal genital exam findings included 6 participants with erythema, 4 with swelling, 2 with warts, 1 with a papular rash, and 3 with skin discoloration.

### STI Test Results

Of the 400 participants, 373 had a vaginal swab collected for GC, CT, or TV testing. Twenty participants did not have a swab collected due to menstruation; 7 did not have a swab collected for other reasons. Forty-nine (13.1%) tested positive for an STI, with CT detected in 41 (11%), GC in 5 (1.3%), and TV in 3 (0.8%) (Table 3). No persons had more than 1 STI. BV was found in 21 (5.6%) of the participants. Concurrent BV was detected among 5 participants with CT, and 2 with GC.

**Table 3:** Vaginal infections and BV diagnosed among AGYW at the enrollment visit.

	**All participants *n* = 373 (%)**	**No prior sex *n* = 297 (%)**	**Prior sex *n* = 76 (%)**	***p* value**
Any STI (combined)^a^	49 (13.1)	33 (11.1)	16 (21.1)	0.022^c^
Chlamydia	41 (11.0)	29 (9.8)	12 (15.8)	0.13^c^
Gonorrhea	5 (1.3)	2 (0.7)	3 (3.9)	0.060^d^
Trichomoniasis	3 (0.8)	2 (0.7)	1 (1.3)	0.50^d^
Bacterial vaginosis	21 (5.6)^b^	10 (3.3)	11 (14.5)	<0.001^c^

*AGYW, adolescent girls and young women; STI, sexually transmitted infection*.

a *Any STI is a combined variable including chlamydia, gonorrhea, and trichomoniasis*.

b *373 participants were tested for chlamydia, gonorrhea, and trichomonas, and 375 participants were tested for bacterial vaginosis*.

c *Chi-squared analysis*.

d *Fisher's exact test*.

### Correlates of STI

Testing positive for an STI did not correlate with age (*p* = 0.46) or whether the participant had a source of income (*p* = 0.29). Among the 297 AGYW who reported no prior sexual activity and had STI testing performed, 33 (11.1%) were diagnosed with either GC, CT, or TV, whereas 16 of 76 (21.1%) AGYW reporting prior sex tested positive (*p* = 0.022). BV positivity also was positively correlated with history of prior sex (*p* <0.001) and testing positive for an STI (*p* = 0.005).

Of the 76 AGYW who reported prior sex and underwent STI testing, 48 (63.2%) reported condom use the first time they had sex. Condom use trended toward an association with a lower STI detection, with 7 of 48 (14.6%) condom users testing positive for an STI, as compared to 9 of 28 (32.1%) AGYW reporting no condom use (*p* = 0.07). Of the 8 AGYW reporting coercion into sex, 7 had STI testing results available; 1 tested positive for CT and 1 for BV. Fifty-nine of 362 AGYW (16.3%) without abnormal vaginal discharge had GC, CT, TV, or BV detected, compared with 4 of 11 (36.4%) of AGYW with abnormal vaginal discharge (*p* = 0.08).

In a logistic regression model of STI predictors, BV, report of prior sex, and vaginal discharge were included in the adjusted model. BV continued to be associated with STI (adjusted OR 1.13, *p* = 0.04, 95% CI 1.01 – 1.27).

## Discussion

In our study of Kenyan AGYW with reported limited sexual experience, more than one in eight persons tested positive for GC, CT, or TV. The most common genital infection was *Chlamydia trachomatis*, which is comparable to results from several studies of adolescents in South Africa (4, 11–13). Prevalence of BV in this cohort, at 5.6%, is much lower than reported in most prior sub-Saharan African cohorts. The low prevalence of BV may be expected, given that our study is among very few reporting STI data from HSV-2 negative and sexually naïve AGYW. Nugent scores were normal in most of our cohort, whereas BV prevalence is markedly higher among other studies of sub-Saharan African women and girls (5). Our findings of extremely low prevalence of BV at study enrollment demonstrates that we may have been successful at enrolling AGYW at the beginning of sexual activity. Given that STIs were observed, our research may indicate that AGYW can go from sexual naivete to engaging in sexual intercourse to acquisition of STIs in a relatively short time frame, indicating a brief window to intervene to reduce risks.

We found that contraceptive use and condom use was low, with heavy reliance on over-the-counter emergency contraceptive pills (EC) as a primary method of family planning. This emphasizes the importance of counseling on and availability of other contraceptive methods, such as long-acting reversible contraceptives.

Many resource-limited countries rely on syndromic management of STIs as per WHO guidelines, in which signs and symptoms associated with an infection direct treatment for the likely causative organism (14). The high prevalence of chlamydia cases in our study underscores the inadequacy of relying on syndromic management, as most chlamydial infections are not symptomatic. The prevalence of CT among youth in the general population is likely even higher, given that our eligibility criteria required minimal prior sexual activity. Diagnostic testing to identify chlamydia and other STIs is urgently needed in this population. This may be difficult to implement, as laboratory-based testing requires expensive resources, facilities, and trained personnel. It can also result in loss-to-follow-up, given the delay in reporting of results. Point-of-care diagnostic testing for STIs is not currently available in Kenya, but should be an active topic of research as it could improve access to screening, provide rapid results, and facilitate timely treatment (15).

Our study was limited by reliance on self-report of sexual activity and risk behaviors, which can lead to recall and social desirability bias. In addition, the questionnaires were administered by clinicians. Participants in the study may have therefore felt pressured to report absence of sexual risk behaviors. The requirement of parental consent for minors may have also been a barrier to accurate reporting. We attempted to mitigate these effects by consenting the parent/guardian separately from the minor, as well as explaining that the data would remain confidential. Our results highlight the challenges of ascertaining behavioral risk factors for AGYW, as most STIs were detected among those who reported no prior sexual intercourse. This further emphasizes how important it is to study this unique and vulnerable population. The current landscape of HIV and STI prevention in sub-Saharan Africa relies on AGYW to self-identify as at risk of these infections to access prevention technology such as pre-exposure prophylaxis. Our research indicates that this may exclude large numbers of at-risk youth. An important next step in creating an AIDS-free generation will be interventions to reach AGYW who may not self-identify or be traditionally categorized as at risk for STI acquisition.

## Data Availability Statement

The datasets generated for this study are available on request to the corresponding author.

## Author Contributions

AR, AW, NM, and KN conceived and planned the study. LO, MM, CK, BC, SS, KN, NM, AW, and AR implemented the study and collected the data. TY, SS, AR, and AM analyzed the data. TY wrote the paper with input from all authors.

## Conflict of Interest

The authors declare that the research was conducted in the absence of any commercial or financial relationships that could be construed as a potential conflict of interest.
